# CNKSR2 gene mutation leads to Houge type of X-linked syndromic mental retardation: A case report and review of literature

**DOI:** 10.1097/MD.0000000000026093

**Published:** 2021-06-11

**Authors:** Qingyun Kang, Liming Yang, Hongmei Liao, Liwen Wu, Bo Chen, Sai Yang, Xiaojun Kuang, Haiyang Yang, Caishi Liao

**Affiliations:** Department of Neurology, Hunan Children's Hospital, Ziyuan Road, Changsha, Hunan, P.R.China.

**Keywords:** CNKSR2, intellectual disability, seizure, whole exome sequencing, X-linked

## Abstract

**Rationale:**

Mutations of connector enhancer of kinase suppressor of Ras-2 (CNKSR2) gene were identified as the cause of Houge type of X-linked syndromic mental retardation. The mutations of CNKSR2 gene are rare, we reporta patient carrying a novel nonsense mutation of CNKSR2,c.625C > T(p.Gln209^∗^) and review the clinical features and mutations of CNKSR2 gene for this rare condition considering previous literature.

**Patient concerns:**

We report a case of a 7-year and 5-month-old Chinese patient with clinical symptoms of intellectual disability, language defect, epilepsy and hyperactivity. Genetic study revealed a novel nonsense variant of CNKSR2, which has not been reported yet.

**Diagnosis:**

According to clinical manifestations, genetic pattern and ACMG classification of mutation site as Class 1-cause disease, the patient was diagnosed as Houge type of X-linked syndromic mental retardation caused by CNKSR2 gene mutation.

**Interventions:**

The patient was administrated with a gradual titration of valproic acid (VPA).

**Outcomes:**

On administration of valproic acid, he had no further seizures.

**Lessons:**

This is the first time to report a nonsense variant in CNKSR2, c.625C > T(p.Gln209^∗^), this finding could expand the spectrum of CNKSR2 mutations and might also support the further study of Houge type of X-linked syndromic mental retardation.

## 1 Introduction

The connector enhancer of kinase suppressor of Ras-2 (CNKSR2) gene, located on chromosome Xp22.12, encodes connector enhancer of KSR2 (CNK2). CNK2 is a multidomain protein playing vital role in synaptic function.^[1,2]^ The mutation in CNKSR2 leads to a broad spectrum of phenotypic variability and manifests as Houge type of X-linked syndromic mental retardation.^[3]^ Here we report a patient with clinical phenotype including global developmental delay, hyperactivity, severe language impairment, and epileptic encephalopathy caused by a nonsense mutation of CNKSR2 (c.625C > T(p.Gln209^∗^)). This nonsense mutation had never been reported, in order to enhance our understanding of the phenotypic and genotypic spectra of CNSKR2 in patients with neurodevelopmental disorders, we report a new patient and retrospectively summarize the 15 previously reported pedigrees.

## 2 Case presentation

### 2.1 Clinical information

**Figure 1:**
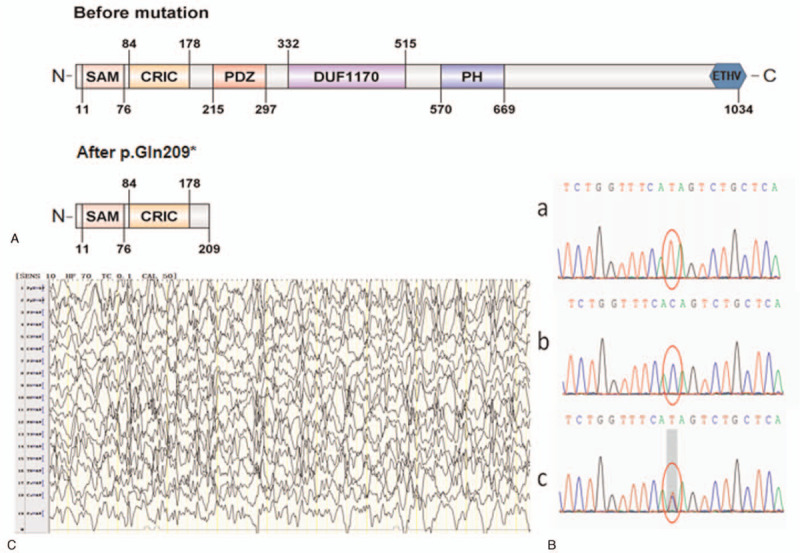
(A). The variant c.625C>T (p.Gln209^∗^) of CNKSR2 leads to a truncated protein without the PDZ, DUF1170, PH, and C-terminal ETHV domains. CRIC = connector enhancer of kinase suppressor of ras, DUF1170 = domain of unknown function, PDZ = PDZ domain, PH = Pleckstrin homology domain, SAM = sterile alpha motif. ETHV motif = Glu-Ser/Thr-Xaa-Val, or E-S/T-X-V in single letter amino acid code (where Xaa/X is any amino acid residue) motif at the COOH terminus. (B). Sequencing of the CNKSR2 gene: c.625C>T (p.Gln209^∗^). The child has a half zygote mutation inherited from his mother, and his father is normal. (a: patient; b: the father; c: the mother). (C). EEG revealed continuous spike-and-slow-waves (CSWS).

A 7-year and 5-month-old Chinese boy was the second child of non-consanguineous, healthy parents. The family history of the patient was unremarkable. The patient was born at 40 weeks of gestation with a birth weight of 3.5 kg, body height of 51 cm and head circumference of 32 cm. His motor development was delayed from birth. He began to gain head and neck stability at 7 months old and rolled over at 11 months old, he can walk alone at the age of 2 years. His language development was severely delayed. At the age of 2 years, he had first epileptic episode lasting for minutes, with eye staring, cyanosis of lip, spitting foam from the mouth, and jerks of the limbs. He was admitted to our department, a four-hour sleep deprived electroencephalogram (EEG) revealed continuous spike-and-slow-waves (CSWS) (Fig. 1C). Cranial magnetic resonance imaging (MRI) and multiple auditory brainstem responses were normal, and no further abnormal findings were noted. As the seizures stopped spontaneously, anti-epileptic therapy was refused by his parents. But four months later, seizures recurred in this patient and then a gradual titration of valproic acid (VPA) was administrated at an initial dose of 10 mg/kg.d increasing to 30 mg/kg.d within one month. On valproic acid of 30 mg/kg.d, he had no further seizures. However, he couldn’t take care of himself or speak until now, and neuropsychological testing show global intellectual disability and hyperactivity.

### 2.2 Molecular genetic analysis

WES and Sanger sequencing were performed by Running Gene Inc. (Beijing, China) using their standard process, which is available in the previous report.^[4]^ In our case, the variant c.625C > T (p.Gln209^∗^) of CNKSR2 was identified (Fig. 1B), that was inherited from his unaffected carrier mother. The mutation located toward the N-terminal of the gene alter the codon sequence of the no. 209 amino acid Gln into a termination codon, resulting in a truncated protein lacking the last 825 of 1034 residues, that leads to a truncated protein without the PDZ, DUF1170, PH, and C-terminal ETHV domains (Fig. 1A). we speculate that the absence of multiple CNKSR2 domains leads to a more detrimental effect on residual protein function(PVS1).Computer-aided analysis predicts that this mutation is harmful (PP3). So far, this mutation has not been reported in our reference population gene database (PM2), so it was classified as ‘pathogenic’ according to the ACMG guidelines. This mutation has been recorded in the Sequence Read Archive (SRA) database and the accession number is SRR14321539.

## 3 Discussion and conclusion

CNKSR2, expressed highly in the brain (especially in the amygdala, cerebellum and hippocampus), plays an important role in Ras signaling-mediated neuronal proliferation, migration and differentiation.^[2]^ The variants or deletions of CNKSR2 affect brain function, leading to seizures and neurodevelopmental disorders.^[5]^ Owing to the patient with CNKSR2-related seizures and neurodevelopmental disorders was first reported by Houge et al,^[3]^ the CNKSR2-related disorders was named as Houge type of X-linked syndromic mental retardation. This genetic syndrome is characterized by intellectual disability, language defect (especially expression of language), epilepsy; attention deficit/ hyperactivity (ADHD), and (CSWS) in early childhood.^[1]^

**Figure 2:**
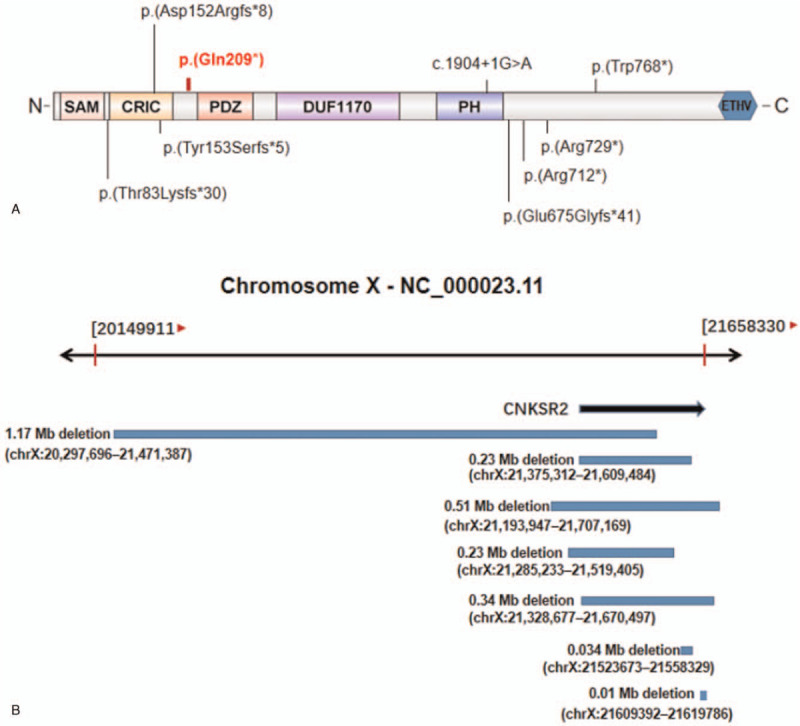
(A). Schematic representation of human CNKSR2 including the positions and the predicted effect of the eight identified variants, p.(Asp152Argfs^∗^8), p.(Arg712^∗^), p.(Arg729^∗^), p.Glu675Glyfs^∗^41, p.Thr83Lysfs^∗^30, p. Tyr153Serfs^∗^5, p.(Trp768^∗^),c.1904+1G>A and p.Gln209^∗^ (this report), in CNKSR2 and on protein level. (B). Genomic map of chromosome Xp22.12 with extent of deletions (blue bars).

Since the first patient with X linkage intelligence disorder associated with CNKSR2 deletions was reported,^[3]^ fourteen more pedigrees with a similar phenotype and different CNKSR2 mutations have been reported. So far, seven kinds of deletions of CNKSR2, either partially or complete, have been reported in a total of nine patients from seven families,^[1,3,6,7]^ there are Xp22.12(21,285,233–21,519,405),Xp22.12(20,297,696–21,471,387), Xp22.12(21,375,312–21,609,484), Xp22.12(21,193,947–21,707,169), Xp22.12(21,328,677–21,670,497), Xp22.12 (21523673–21558329) and Xp22.12 (21609392–21619786) (Fig. 2A). In addition, eight kinds of variants in CNKSR2 had been reported in the literature,^[1,5,7–10]^ there are c.452insA(p.Asp152Argfs^∗^8), c.2024_2027delAGAG (p.Glu675Glyfs^∗^41), c.246–247delAG(p.Thr83Lysfs^∗^30), c.457_461del(p. Tyr153Serfs^∗^5), c.2314 C > T(p.Arg712^∗^), c.2185C > T(p.Arg729^∗^), c.2304G > A (p.Trp768^∗^) and c.1904+1G > A (Fig. 2B).Among the twenty one cases reported, nine of them have their genotype being inherited from mothers(Table 1). Because of the X-inactivation in females, most of carrier mothers had normal phenotype. Compared with those patients reported, herein we identified a novel CNKSR2 (c.625C>T (p.Gln209^∗^))truncating variant in a Chinese boy. In our case, this variant c.625C>T (p.Gln209^∗^) was inherited from his unaffected carrier mother.

**Table 1:** Comparison of reported pedigrees and cases with CNKSR2 gene variants.

Pedigree no	Publication	Gender	CNKSR2 variant	Occurrence	intellectual disability	Language defect	Attention problems	MRI Scanning	Seizure	Seizure onset-age	Seizure outcome	CSWS	Female carrier
1	Houge et al ^[3]^	male	Deletion Xp22.12 (21,285,233–21,519,405)	Maternal	ID of mild to moderate	Yes	Yes	N/A	Yes	N/A	seizure free	N/A	Normal
2 (two siblings)	Vaags et al ^[1]^	male	Deletion Xp22.12 (20,297,696–21,471,387)	Maternal	Yes	Yes	Yes	Normal	Yes	2y	N/A	Yes	Mild learning disability
					Yes	Yes	Yes	Normal	Yes	2y3m	seizure free	Yes	
3	Vaags et al ^[1]^	male	Deletion Xp22.12 (21,375,312–21,609,484)	N/A	Yes	Yes	Yes	Normal	Yes	2y6m	seizure free	Yes	N/A
4 (two siblings)	Vaags et al ^[1]^	male	Deletion Xp22.12 (21,193,947–21,707,169)	N/A	Yes	Yes	Yes	Nonspecific periventricular white matter hyperintensity	Yes	8d	seizure free	No	N/A
				N/A	Yes	Yes	Yes	Normal	No	/	/	No	N/A
5 (three siblings)	Vaags et al ^[1]^	male	c.452insA (p.Asp152Argfs^∗^8)	N/A	Yes	Yes	Yes	minor cortical atrophy	Yes	N/A	seizure free	N/A	N/A
				N/A	Yes	Yes	Yes	Normal	Yes	N/A	seizure free	N/A	N/A
				N/A	Yes	Yes	Yes	Normal	Yes	N/A	seizure free	N/A	N/A
6	Aypar et al ^[6]^	male	Deletion Xp22.12 (21,328,677–21,670,497)	Maternal	Yes	Yes	N/A	Normal	Yes	N/A	No	Yes	Normal
7 (three siblings)	Damiano et al ^[5]^	male	c.2314 C>T(p.Arg712^∗^)	Maternal	Yes	Yes	Yes	N/A	Yes	3y6m	N/A	Yes	febrile seizures
		male			Yes	Yes	Yes	N/A	Yes	3y6m	N/A	Yes	
		female			mild motor and language delay	NA	NA	N/A	Yes	6y	seizure free	N/A	
8	Sun et al ^[8]^	male	c.2185C>T (p.Arg729^∗^)	De novo	Yes	Yes	Yes	Normal	Yes	∼ 2y	Improvement	Yes	/
9	Zhang et al ^[10]^	male	c.1904+1G>A	Maternal	mild	No	Yes	Normal	No	/	/	No	Mild learning disability
10	Polla et al ^[9]^	female	c.2304G>A (p.Trp768^∗^)	De novo	Yes	No	No	Normal	Yes	NA	N/A	N/A	/
11	Bonardi et al ^[7]^	male	c.2024_2027delAGAG (p.Glu675Glyfs^∗^41)	De novo	Yes	Yes	N/A	Normal	Yes	2y	Multiple daily atypical absences	Yes	/
12	Bonardi et al ^[7]^	male	c.246–247delAG (p.Thr83Lysfs^∗^30)	De novo	Yes	Yes	Yes	Normal	Yes	3y	seizure free	Yes	/
13	Bonardi et al ^[7]^	male	c.457_461del(p. Tyr153Serfs^∗^5)	Maternal mosaicism (5/156 reads)	Yes	Yes	N/A	Normal	Yes	4y	Atypical absences with head drops	Yes	Normal
14	Bonardi et al ^[7]^	female	Deletion Xp22.12 (21523673–21558329)	Parents unavailable	Yes	Yes	Yes	Normal	Yes	6y	seizure free	Yes	/
15	Bonardi et al ^[7]^	male	Deletion Xp22.12 (21609392–21619786)	De novo	Yes	Yes	Yes	Normal	Yes	3y2m	9y8m: 1–2seizures/year	Yes	/
16	our case	male	c.625C>T(p.Gln209^∗^)	Maternal	Yes	Yes	Yes	Normal	Yes	2y	seizure free	Yes	Normal

Except one patient with splicing variant, all previously reported male patients exhibited seizures and language impairment. Continuous spike-and-slow-waves (CSWS) on EEG has been documented in twelve reported cases, consistent with the patient in our study. Intellectual disability and psychomotor delay were also recognized in all of patients reported previously, other common and uncharacteristic features including severe attention deficit and hyperactivity have been frequently observed in the literature (Table 1). The patient in our study had a consistent clinical phenotype with the previous descriptions. He showed intellectual disability, language defect, epilepsy and hyperactivity. Therefore, combined with our patient's clinical manifestations, genetic pattern and ACMG classification of mutation site as Class 1-cause disease, the patient was diagnosed as Houge type of X-linked syndromic mental retardation caused by CNKSR2 gene mutation. He had onset of seizures at 2 years, developmental delay existed prior to the onset of seizures. After the treatment of valproic acid, he had no further seizures. Even though the patient was free from seizures, he still had psychomotor delay and language. The intellectual disability was severe. And the patient couldn’t speak until now. This is consistent with the finding that CNKSR2 is expressed prenatally, suggesting a vital role in neurodevelopment.

In conclusion, we reported a case of CNKSR2 c.625C>T (p.Gln209^∗^) nonsense variants in a child with Houge type of X-linked syndromic mental retardation. This site is the first to be identified, which broadens the spectrum of genetic variants of this gene. We hope that our research can improve clinicians’ understanding of the disease.

## Acknowledgments

We would like to thank the patient and his family for their cooperation. We also appreciat Running-Gene Inc. for professional genetic sequencing.

## Author contributions

**Conceptualization:** Liming Yang.

**Data curation:** Sai Yang, Xiaojun Kuang.

**Formal analysis:** Hongmei Liao, Haiyang Yang, Caishi Liao.

**Supervision:** Liming Yang, Liwen Wu, Bo Chen.

**Writing – original draft:** Qingyun Kang.

**Writing – review & editing:** Qingyun Kang.
